# Metabolic dependencies and neural progenitor dysregulation: driving forces in paediatric high-grade glioma development

**DOI:** 10.1007/s10555-026-10325-2

**Published:** 2026-03-13

**Authors:** Yan Hay Grace Lee, Maria Tsoli, Yan Chuan Shi, Chi Kin Ip, David Ziegler

**Affiliations:** 1 Children’s Cancer Institute at Minderoo Children’s Comprehensive Cancer Centre, Sydney, NSW, Australia, Sydney, NSW Australia; 2 School of Clinical Medicine, UNSW Medicine and Health, Sydney, NSW Australia; 3 Faculty of Medicine, University of New South Wales, Sydney, NSW 2052 Australia; 4 Brain & Body Metabolism Lab, Victor Chang Cardiac Research Institute, Lowy Packer Building, 405 Liverpool Street, Darlinghurst, NSW 2010 Australia; 5 Kid’s Cancer Centre, Sydney Children’s Hospital, Randwick, NSW Australia

**Keywords:** Paediatric high-grade gliomas, Tumour microenvironment, Neural progenitor cells

## Abstract

Paediatric high-grade gliomas (pHGGs) are the most lethal brain tumours in children, characterised by profound epigenetic dysregulation and limited treatment options. The 2021 WHO Classification has established a molecular framework that distinguishes pHGGs as biologically distinct from adult glioblastoma, recognising four major subtypes: H3K27-altered diffuse midline glioma, H3G34-mutant diffuse hemispheric glioma, infant-type hemispheric glioma, and the rare IDH-mutant gliomas. Each subtype exhibits unique epigenetic landscapes, metabolic dependencies, and therapeutic vulnerabilities, necessitating subtype-specific treatment strategies. This review explores the molecular classification of pHGGs and examines the critical role of the tumour microenvironment in disease progression. We focus on glioma stem cells as central drivers of tumour initiation, maintenance, and therapeutic resistance, highlighting their remarkable cellular plasticity and ability to dynamically transition between different states. Particular attention is given to metabolic reprogramming in pHGGs, including alterations in glucose and lipid metabolism, and the exceptional metabolic flexibility of glioma stem cells that enables adaptation to microenvironmental pressures. Importantly, we discuss the intimate crosstalk between metabolism and epigenetic regulation, whereby metabolites serve as essential cofactors for chromatin-modifying enzymes—exemplified by α-ketoglutarate maintaining low H3K27me3 in H3K27M tumours and 2-hydroxyglutarate driving hypermethylation in IDH-mutant gliomas. We review preclinical models that have advanced pHGG research and discuss emerging immunotherapeutic approaches, including CAR T-cell therapies and oncolytic viruses. By synthesising current understanding of pHGG biology, this review aims to identify promising therapeutic avenues that exploit the unique metabolic and epigenetic vulnerabilities of each molecular subtype.

## Introduction

Paediatric high-grade gliomas (pHGGs) represent the most aggressive form of childhood brain tumours, with currently no effective treatment options, and the disease remains palliative [1]. The hallmark of pHGGs lies in their profound epigenetic dysregulation, leading to widespread disturbances in gene expression that drive oncogenesis. Like any other tumours, pHGGs are highly heterogeneous, with distinct epigenetic drivers and transcriptional signatures existing across different molecular subtypes, highlighting the importance of targeted therapies for individual [2]. According to the 2021 WHO Classification of Tumours of the Central Nervous System, paediatric-type diffuse high-grade gliomas are now recognised as molecularly and biologically distinct entities from adult glioblastoma, which is specifically reserved for adult IDH-wildtype tumours [3]. This distinction reflects fundamental differences in cells-of-origin, developmental contexts, molecular drivers, and therapeutic vulnerabilities between paediatric and adult tumours. Unfortunately, there is no cure for pHGGs. Standard interventions such as surgical resection, chemotherapy, and radiotherapy are initiated upon diagnosis to slow disease progression, but clinical benefit is modest. Effectiveness remains limited by the highly aggressive nature of these tumours, their frequent location in surgically inaccessible regions such as the brainstem, and the shortage of subtype-specific therapeutic targets [4].

Beyond intrinsic genetic and epigenetic abnormalities, the tumour microenvironment (TME) has emerged as a critical driver of pHGGs development and therapy resistance. The TME is composed of a complex network of various components, including vasculature, immune cells, neurons, and glioma stem cells (GSCs) [5]. Recent *in vivo* studies suggest that GSCs may act as the cell of origin of pHGGs, with their extensive self-renewal capability contributing to glioma initiation and progression [6, 7]. Despite advances in molecular profiling, the biological mechanisms that underlie tumour maintenance and resistance remain poorly understood, particularly regarding the role of GSCs in shaping the tumour niche, their remarkable cellular plasticity in response to microenvironmental cues, and their contribution to therapeutic failure. In addition, glioma cells exhibit profound metabolic reprogramming to support their elevated energy demands and biosynthetic activity [8]. These metabolic alterations not only fuel tumour growth and survival but also contribute to treatment resistance. Notably, GSCs exhibit extensive metabolic plasticity that facilitates glioma maintenance and fosters disease progression. Moreover, emerging evidence reveals that metabolism and epigenetic regulation are intimately interconnected, with metabolites serving as essential cofactors for chromatin-modifying enzymes that fundamentally shape cellular identity and therapeutic responses across pHGG molecular subtypes.

In this review, we outline the molecular subtypes of pHGGs according to the WHO 2021 classification and explore key players within the TME that influence glioma development. We examine the multifaceted roles of glioma stem cells in tumour development, maintenance, and therapy resistance, with particular emphasis on cellular plasticity and dynamic state transitions. We then discuss metabolic reprogramming in pHGGs, focussing on alterations in glucose and lipid metabolism, the metabolic flexibility of GSCs in adapting to their dynamic microenvironment, and the critical interplay between metabolic and epigenetic regulation. Additionally, we review preclinical models that have enabled advances in pHGG research and discuss emerging immunotherapeutic approaches including CAR T-cell therapies and oncolytic viruses. By synthesising recent research findings, this review aims to provide insights into the pathophysiology of pHGGs and identify promising avenues for subtype-specific therapeutic intervention.

## Molecular classification of paediatric-type diffuse high-grade gliomas

Recent advances in molecular profiling have revolutionised pHGG classification, moving beyond histological grading to molecular definitions that better reflect tumour biology and clinical behaviour. The WHO 2021 classification delineates four major molecular categories of paediatric-type diffuse high-grade gliomas: (i) diffuse midline glioma, H3K27-altered; (ii) diffuse hemispheric glioma, H3G34-mutant; (iii) diffuse paediatric-type high-grade glioma, H3-wildtype and IDH-wildtype; and (iv) infant-type hemispheric glioma^24^. Additionally, IDH-mutant gliomas, though exceedingly rare in paediatric populations, are recognised as a distinct entity^24^. This molecular-based classification reflects distinct cells-of-origin, developmental contexts, and therapeutic vulnerabilities, enabling development of subtype-specific targeted therapies.

### Diffuse midline glioma, H3K27-altered

Histone H3 proteins are key components of chromatin, essential for DNA condensation and nucleosome formation [9]. Beyond their structural role, they regulate gene expression through various chemical modifications that influence chromatin accessibility. The N-terminal tails of histone H3 are subject to diverse post-translational modifications—such as methylation, acetylation, phosphorylation, and ubiquitination—which collectively form the “histone code” that governs chromatin accessibility and the activation or repression of gene transcription [9]. Recurrent mutations in the histone H3 genes, particularly H3F3A and HIST1H3B, have been identified as key drivers in pHGG development [3, 10, 11].

The H3K27M mutation, characterised by substitution of lysine (K) with methionine (M) at position 27 of histone H3.1 (HIST1H3B) or H3.3 (H3F3A), accounts for approximately 80% of diffuse midline gliomas [12]. Previously known as diffuse intrinsic pontine glioma (DIPG), these tumours are now classified under the WHO 2021 nomenclature as diffuse midline glioma, H3K27-altered. Biochemically, the H3K27M mutation exerts a dominant-negative effect on Polycomb Repressive Complex 2 (PRC2) activity, resulting in global loss of trimethylation at lysine 27 (H3K27me3) and driving aberrant transcription that promotes tumourigenesis [11, 13]. This epigenetic disruption fundamentally alters the chromatin landscape, leading to dysregulated gene expression programmes that favour oncogenic transformation [11].

Diffuse midline gliomas predominantly develop in midline structures including the pons, thalamus, spinal cord, and other central brain regions, with the pons being the most common site in paediatric patients (Table 1) [3, 10]. These aggressive tumours primarily affect children between 5 and 11 years of age, with a peak incidence at 6–8 years. The clinical prognosis remains universally poor, with median overall survival of only 9–11 months and 2-year survival rates below 10% (Table 1). Key immunohistochemical markers include loss of H3K27me3 expression and positive staining for OLIG2 and GFAP (Table 1) [3, 11]. Radiologically, these tumours typically appear as T1 hypointense and T2/FLAIR hyperintense lesions with diffuse pontine involvement and minimal or no contrast enhancement (Table 1) [14].

**Table 1:** Clinical classification summary of the major molecular categories of paediatric diffuse high-grade glioma subtypes, including WHO 2021 classification, key molecular alterations, typical location, age at diagnosis, radiological features, and prognostic factors

WHO 2021 classification	Key molecular alterations	Locations	Age at diagnosis	Median OS (months)	Radiological features	Key IHC markers	Prognostic factors & outcome	Reference
Diffuse midline glioma, H3K27-altered	H3K27M (H3F3A or HIST1H3B); TP53, ACVR1, PDGFRA alterations	Pons, thalamus, spinal cord, other midline structures	5–11 years (peak 6–8)	< 12	T1 hypointense, T2/FLAIR hyperintense; diffuse pontine involvement; minimal/no enhancement	Loss of H3K27me3, OLIG2 +, GFAP + (variable)	Universally poor (2-year OS < 10%); infratentorial location associated with worse outcome; multimodal therapy (surgery + radiation) improves OS; ACVR1 co-mutation associated with younger age and slightly better survival	[3, 10–17]
Diffuse hemispheric glioma, H3G34-mutant	H3G34R/V (H3F3A), TP53, ATRX alterations	Cerebral hemispheres (frontal, parietal, temporal lobes)	15–25 years (older children/young adults)	< 24	Heterogenous enhancement; cortical/subcortical location; cystic changes common	OLIG2 +, GFAP + (variable), retained H3K27me3, loss of ATRX	Poor to intermediate (2-year OS ~ 39%); favourable factors: female sex, gross total resection, MGMT methylation (75% cases), older age ($\ge$ 18 years); unfavourable factors: pial invasion, G34V (vs G34R) mutation	[3, 13, 18–23]
Diffuse paediatric-type HGG, H3-wildtype and IDH-wildtype	EGFR amplification, CDK4/6 amplification, MYCN amplification, PDGFRA amplification, RTK alterations	Variable; cerebral hemisphere or throughout CNS	Variable (infancy to adolescence)	Variable (6–24 depending on subgroup)	Heterogeneous; depends on genetic subgroup	OLIG2 +, GFAP +, retained H3K27me3	Highly variable by molecular subgroup; RTK-altered (BRAF V600E) relatively favourable; MYCN-amplified poorest outcome; EGRF-amplified intermediate	[41, 42]
Infant-type hemispheric glioma	Receptor tyrosine kinase (RTK) fusions (ALK, ROS1, NTRK, MET); no H3 or IDH mutations	Cerebral hemispheres	< 18 months	24 to 36 +	Large, well-circumscribed; solid-cystic; variable enhancement	OLIG2 +, GFAP + (variable), retained H3K27me3	Relatively favourable compared to other pHGGs (2-year OS ~ 74%); excellent response to target therapy (TRK/ALK inhibitions); ALK-fusions better than ROS1-fushions; extent of resection impact outcome	[35–40]
IDH-mutant glioma (rare in paediatrics)	IDH1/2 mutations	Cerebral hemispheres (frontal, temporal lobe)	Adolescence/young adults (rare < 15 years)	24 to 36 +	Variable enhancement; often in frontal lobes	IDH1 R132H +, loss of ARTX, retained H3K27me3	Better than IDH-wildtype but still aggressive in paediatrics; MGMT methylation status impacts treatment response; extent of resection is an important prognostic factor	[3, 24–34]

Despite advances in neurosurgery, radiotherapy, and chemotherapy, treatment options remain primarily palliative, with radiation therapy representing the current standard of care [15]. Prognostic factors include tumour location, with infratentorial lesions demonstrating worse outcomes compared to supratentorial counterparts, and the presence of ACVR1 co-mutations, which are associated with younger age at diagnosis and marginally improved survival (Table 1) [10, 12]. Ongoing research focuses on identifying targetable molecular vulnerabilities, including epigenetic modifiers such as EZH2 and HDAC inhibitors, as well as immunotherapeutic approaches [10, 16]. Moreover, the blood–brain barrier represents a significant challenge in delivering therapeutics to brainstem tumours, restricting clinical application of many promising anti-cancer agents. Current clinical trials are exploring novel molecular targets, convection-enhanced delivery systems, and focussed ultrasound techniques to enhance drug penetration while minimising systemic toxicity [17].

### Diffuse hemispheric glioma, H3G34-mutant

Diffuse hemispheric glioma, H3G34-mutant represents a distinct molecular entity within paediatric-type diffuse high-grade gliomas, characterised by missense mutations in the H3F3A gene resulting in substitution of glycine with either arginine (G34R, most common) or valine (G34V) at position 34 of the histone H3.3 protein [3, 18]. This mutation leads to steric hindrance that prevents di- and tri-methylation of lysine 36 (H3K36), thereby disrupting posttranslational modifications critical for glial differentiation and establishing an oncogenic chromatin landscape [18, 19]. Unlike the H3K27M mutation which causes global epigenetic changes, the H3G34R/V mutation exerts more localised effects on gene regulation, contributing to its distinct clinical and biological profile [18, 19].

Diffuse hemispheric gliomas predominantly affect older children, adolescents, and young adults, with a median age at diagnosis of 15–16 years, representing a markedly older demographic compared to H3K27-altered diffuse midline gliomas (Table 1) [13, 20, 21]. These tumours arise in the cerebral hemispheres, most commonly involving the frontal, parietal, and temporal lobes, with a characteristic cortical and subcortical distribution (Table 1) [20, 22]. Radiologically, diffuse hemispheric gliomas demonstrate heterogeneous contrast enhancement with frequent cystic changes, distinguishing them from the minimally enhancing diffuse midline gliomas (Table 1). Histologically, these tumours often display high-grade features with either astrocytic or primitive embryonal-like morphology.

The molecular landscape of H3G34-mutant gliomas is characterised by nearly universal co-occurrence of TP53 mutations (88%) and ATRX loss (93%), alongside PDGFRA alterations and frequent MGMT promoter methylation (75% of cases) (Table 1). Immunohistochemically, these tumours are positive for OLIG2 and variably positive for GFAP, while crucially demonstrating retained H3K27me3 expression (in contrast to H3K27-altered gliomas) and loss of ATRX nuclear staining (Table 1) [19, 20].

The clinical prognosis for diffuse hemispheric glioma remains poor but intermediate compared to diffuse midline gliomas, with median overall survival ranging from 17–22 months to 2-year survival of approximately 39% (Table 1). Several prognostic factors have been identified through systematic analyses. Favourable prognostic indicators include female sex, gross total resection, MGMT promoter methylation, and age ≥ 18 years at diagnosis (Table 1) [13, 21]. Conversely, unfavourable factors include pial invasion and the G34V mutation variant, which demonstrates significantly shorter survival compared to G34R mutations (Table 1). Notably, time to progression averages 10 months, with a median interval of 5 months from progression to death, emphasising the aggressive nature of recurrent disease.

Current treatment strategies involve maximal safe surgical resection followed by radiation and chemotherapy, though optimal therapeutic regimens remain under investigation [13, 20]. The high frequency of PDGFRA mutations and CDK6 amplifications in these tumours has sparked interest in targeted therapies, including PDGFR inhibitors and CDK4/6 inhibitors, though clinical validation remains limited [19, 23]. Despite representing a distinct molecular entity since the 2021 WHO classification, research on H3G34-mutant gliomas remains substantially less extensive than studies on H3K27-altered tumours, underscoring the critical need for dedicated research efforts to better understand the pathogenesis of this subtype and develop subtype-specific therapeutic strategies.

### IDH-mutant gliomas (rare in paediatrics)

IDH-mutant gliomas are exceedingly rare in the paediatric population, occurring primarily in adolescents and young adults rather than children under 15 years of age (Table 1) [3, 24, 25]. When present, these tumours typically arise in the frontal and temporal lobes of the cerebral hemispheres (Table 1). IDH genes encode isocitrate dehydrogenase enzymes that catalyse the conversion of isocitrate to α-ketoglutarate in the tricarboxylic acid cycle [24]. Mutations in IDH1 (most commonly R132H) or IDH2 confer a neomorphic function, whereby the mutant enzyme aberrantly produces the oncometabolite 2-hydroxyglutarate (2-HG) [26, 27]. Accumulation of 2-HG inhibits α-ketoglutarate-dependent dioxygenases, resulting in widespread DNA and histone hypermethylation that promotes gliomagenesis [28, 29].

Immunohistochemically, these tumours are characterised by positive staining for IDH1 R132H mutant protein, loss of ATRX expression, and retained H3K27me3 (Table 1) [30, 31]. Despite their high-grade features, IDH-mutant gliomas demonstrate better prognosis than IDH-wildtype counterparts, with median overall survival of 24 to 36 + months (Table 1) [32]. Important prognostic factors include extent of surgical resection and MGMT promoter methylation status (Table 1) [33]. Treatment involves maximal safe resection followed by radiation and temozolomide chemotherapy, with ongoing trials investigating specific IDH inhibitors [33, 34].

### Infant-type hemispheric glioma

Infant-type hemispheric glioma is a molecularly distinct entity predominantly affecting infants under 18 months of age, characterised by gene fusions involving receptor tyrosine kinases (RTKs) including ALK, ROS1, NTRK1/2/3, and MET [35, 36]. These RTK fusions occur in up to 57.5% of infant high-grade gliomas as single driving events, without the histone H3 or IDH mutations that define other pHGG subtypes [37].

Unlike other pHGGs, infant-type hemispheric gliomas demonstrate a favourable prognosis with 2-year overall survival rates approaching 74%, particularly when treated with appropriate targeted therapies [35]. FDA-approved TRK inhibitors (larotrectinib, entrectinib) and ALK inhibitors (lorlatinib) have shown remarkable efficacy in these tumours, with response rates of 75% and dramatic radiographic responses documented within months of therapy initiation [38–40]. This represents a paradigm shift towards precision medicine in paediatric neuro-oncology, where molecular profiling guides targeted treatment strategies and avoids the neurocognitive sequelae associated with cranial radiation in this vulnerable population.

### Diffuse paediatric-type high-grade glioma, H3-wildtype, and IDH-wildtype

H3/IDH-wildtype pHGGs represent a rare and molecularly heterogeneous group with highly variable clinical outcomes (Table 1). Molecular profiling identifies three major subgroups: (i) RTK-altered tumours (BRAF V600E, NF1) with relatively favourable prognosis and responsiveness to BRAF/MEK inhibitors, (ii) EGFR/CDK4/6/MYCN-amplified tumours with the poorest outcomes and widespread CNS infiltration, and (iii) PDGFRA/MET-amplified tumours with intermediate prognosis (Table 1) [41, 42].

Median survival ranges from 6–24 months depending on molecular subgroup (Table 1). Immunohistochemistry shows OLIG2 +, GFAP +, and retained H3K27me3 (Table 1) [41, 42]. The variable prognosis underscores the critical importance of molecular profiling for risk stratification and treatment selection.

Despite the genetic classification of pHGGs mutants, critical gaps remain in our understanding of how these tumours develop within the brain. These tumours are surrounded by non-malignant cells that create a favourable microenvironment sustaining their aggressive growth. The spatial organisation of these cellular components further modulates tumour behaviour, introducing an additional layer of heterogeneity beyond genetic subtypes. [5]

## Metabolic heterogeneity across pHGG molecular subtypes

Having established the distinct molecular entities within paediatric-type diffuse high-grade gliomas, it is critical to determine how these molecular classifications translate into differential metabolic dependencies. Recent single-cell RNA sequencing and spatial transcriptomics have revealed that metabolic reprogramming varies substantially across pHGG subtypes, with each molecular entity exhibiting unique metabolic landscapes [43–47]. While comprehensive metabolic profiling remains limited for certain entities, H3K27M-altered diffuse midline gliomas, H3G34-mutant diffuse hemispheric gliomas, and IDH-mutant gliomas have been most extensively characterised, revealing distinct metabolic vulnerabilities that may inform subtype-specific therapeutic strategies [43, 44].

H3K27M-altered diffuse midline gliomas exhibit profound metabolic reprogramming characterised by enhanced glycolysis, glutaminolysis, and tricarboxylic acid (TCA) cycle metabolism [43]. Integrated analysis combining patient-derived cell lines, bulk and single-cell RNA-sequencing, and *in vivo* magnetic resonance spectroscopy demonstrates that H3K27M tumours show significantly elevated expression of key metabolic enzymes, including glucose transporter SLC2A3/GLUT3, hexokinase 2 (HK2), and glutamate dehydrogenase (GDH) [43, 44]. These metabolic alterations generate high levels of α-ketoglutarate (α-KG), which helps maintain the characteristic low H3K27me3 levels in these tumours. Inhibition of key glycolytic or glutaminolytic enzymes increases H3K27me3, alters chromatin accessibility, and prolongs survival in preclinical models [43].

Single-cell RNA-sequencing reveals substantial metabolic heterogeneity within individual H3K27M tumours [44, 45]. Less-differentiated oligodendrocyte precursor cell (OPC)-like cells—which constitute the tumour majority and exhibit greater tumour-propagating potential—display enhanced glycolytic activity [45, 46]. In contrast, more differentiated astrocyte (AC)-like cells demonstrate increased ferroptosis sensitivity, while OPC-like cells exhibit diminished mitochondrial oxidative phosphorylation and heightened sensitivity to cholesterol biosynthesis inhibitors [46]. These findings highlight that cellular differentiation state dictates distinct metabolic vulnerabilities, providing opportunities for state-specific therapeutic targeting. Moreover, paediatric gliomas including H3K27M-altered tumours demonstrate enriched glucose availability with reduced lactate accumulation compared to adult gliomas, which preferentially utilise fatty acid oxidation [47].

IDH-mutant gliomas, though rare in paediatric populations, demonstrate a fundamentally distinct metabolic profile driven by the oncometabolite 2-hydroxyglutarate (2-HG) [26, 27]. As discussed previously, the neomorphic IDH1/2 mutations result in aberrant conversion of α-ketoglutarate to 2-HG, which competitively inhibits α-ketoglutarate-dependent dioxygenases and establishes widespread epigenetic dysregulation [28]. This metabolic-epigenetic axis represents a targetable vulnerability distinct from H3-mutant subtypes.

In contrast, metabolic profiling of H3G34-mutant diffuse hemispheric gliomas remains substantially less comprehensive. Proteomic studies suggest that H3G34R mutations alter mitochondrial metabolism [18], though detailed single-cell metabolic characterisation is lacking. Similarly, the diverse molecular subgroups within H3/IDH-wildtype pHGGs likely harbour distinct metabolic dependencies corresponding to their unique oncogenic drivers (EGFR, MYCN, RTK alterations), yet systematic metabolic profiling across these subgroups remains incomplete [41, 42]. These gaps represent critical priorities for future investigation, as subtype-specific metabolic vulnerabilities may provide tractable therapeutic targets where conventional therapies have failed.

## Tumour microenvironment in pHGGs development

Apart from epigenetic aberrations, a growing body of evidence demonstrates that the tumour microenvironment (TME) plays a critical role in driving pHGGs development. The TME is a complex and dynamic oncogenic-favourable network comprising highly heterogeneous cell types, including endothelial cells, neurons, neural stem cells (NSCs), astrocytes, oligodendrocytes, pericytes, and microglia, all of which can contribute to tumour growth. These diverse cellular components provide a unique tumour milieu, featuring highly vascularised networks, fully functional neural-tumour connections, stem cell niches, and a distinct immunological landscape [5]. While most of the immune components in the TME have been extensively studied, the functional contributions of neurons and NSCs in supporting tumour initiation and development remain largely unknown [48, 49]. This section will discuss the relevant literature reporting the functional interactions between each TME cell population and tumour cells, highlighting their complex relationships and implications for cancer development.

### Complex vascular system within pHGGs

Angiogenesis is a critical process for glioma cells to recruit excess nutrients and oxygen for themselves and infiltrate surrounding healthy tissues. Pathologically, cancerous vessels exhibit distinctive morphological alterations, including cellular structural reorganisation, disruption of tight junctions, increased perivascular space, and deficiency in pericyte coverage [50–52]. Unlike normal vessels, tumour vessels constantly proliferate and expand from existing tumour sites, forming a leaky and haemorrhagic vascular network [53]. This compromises the BBB, inducing hypoxia and necrosis in tumour tissues. Intriguingly, the vascular architecture and BBB integrity vary across pHGGs subtypes. Wei et al. [54] demonstrated there are subtype-specific vascular differences in PDX mouse models, with DIPG exhibiting a relatively intact and less permeable BBB compared to DHG. This difference may stem from the tumour’s anatomical location, as DIPG arises in the brainstem, a region with a more robust BBB and fewer fenestrations compared to the cerebral hemispheres, the typical site of DHG.

During early glioma development, the BBB remains intact, and the tumour relies on normal brain vessels. As the glioma progresses, endothelial cells detach from normal vessels and form new angiogenic spots near the tumour [51]. Within the tumourigenic microenvironment, the normal regulation of angiogenesis is impaired, favouring the expression of pro-angiogenic genes, particularly VEGF to foster communication between the tumour and endothelial cells [53, 55]. Therefore, the limited permeability of the BBB in DIPG raises additional therapeutic challenges, limiting effective delivery routes. 

### Interactions of immune cells and pHGGs

The complex immune responses in pHGGs arise from the interplay between glioma cells and immune cells [56]. Studies using immunohistochemistry reveal elevated levels of tumour-associated macrophages (TAM) and T-cells in pHGGs compared to normal brain tissue. However, the level of infiltrating T-cells varies across pHGG subtypes, with DIPG cells showing limited T-cell infiltration [57]. This scarcity reflects a failure of immunosurveillance in DIPG for tumour elimination. In addition, NanoString transcript analysis of immune-cell specific chemokine genes confirmed an inflammatory microenvironment in pHGGs with significant TAM infiltration around necrotic areas and heightened levels of the inflammatory cytokine CXCL8 and the immunosuppressive factor programmed death ligand 1 (PD-L1) within the tumour [57], however, such responses fail to target cancer cells.

Microglia are the primary resident immune cells of the CNS. Microglia within the tumour microenvironment exhibit significant plasticity, displaying a dynamic activation state with a heterogeneous mix of M1 and M2 markers that contribute to tumour growth and immune suppression [56, 58]. Research has revealed that specific epigenetic changes in histone deacetylases 5 and 9 (HDAC5 and HDAC9) push microglia toward an M2 phenotype. This M2 state reduces inflammatory responses and helps tumours evade the immune system. However, contradictory evidence exists, as other studies have found that M1-specific markers and their associated signalling pathways are also positively linked with glioma progression and growth [59, 60]. Glioma cells influence microglia through multiple pathways. First, glioma-induced microglial activation enhances PD-1/PD-L1 binding, which suppresses anti-tumour immune responses. In addition, gliomas cause overexpression of the CCL2 chemokine receptor, which modifies the CCR2 signalling pathway, leading to excessive microglial recruitment to the tumour site. Both mechanisms ultimately promote gliomagenesis and immune evasion, though they operate through different molecular pathways [61, 62].

### Interactions of neurons and pHGGs

**Fig. 1:**
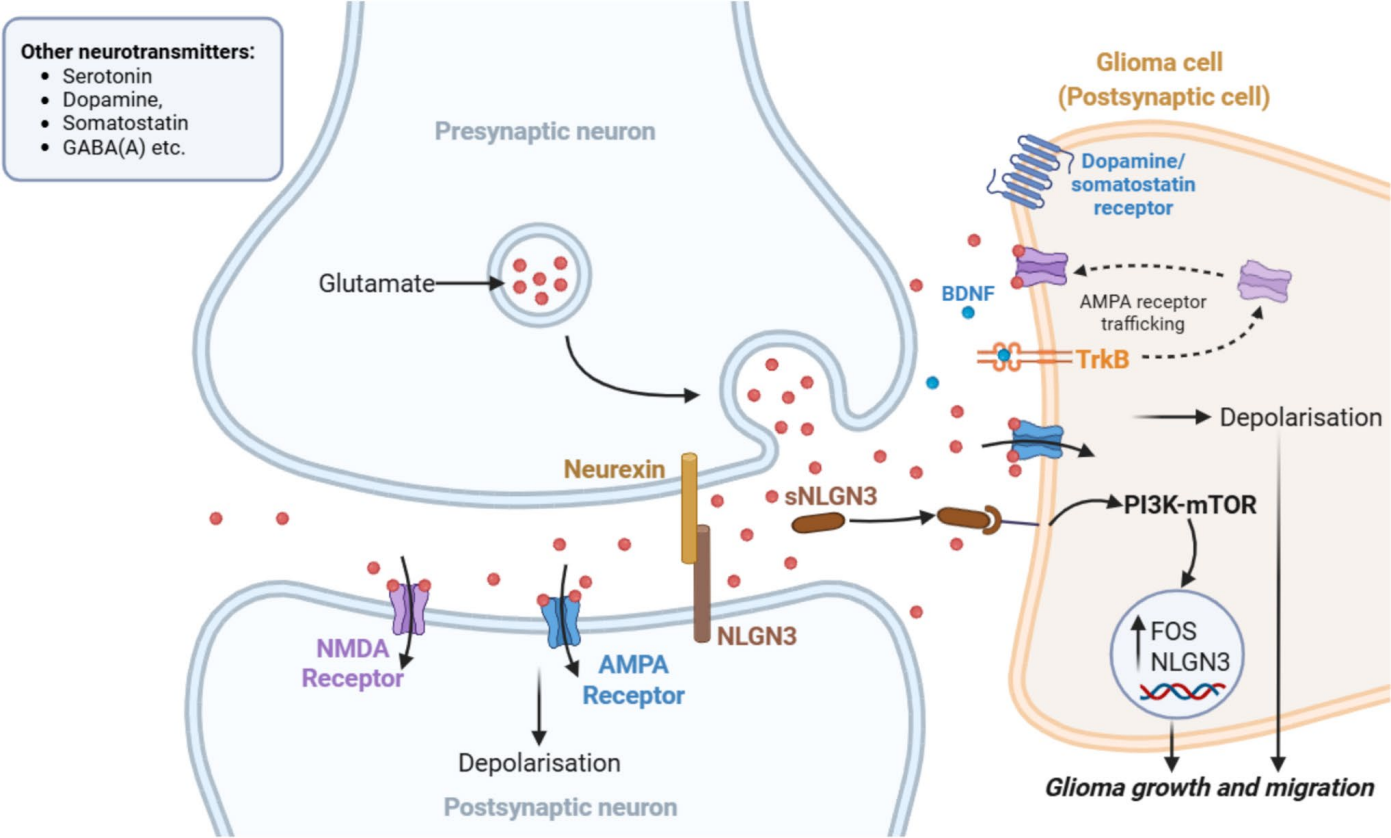
Synapse formation of cancer cells and neurons. Neuron communication typically via glutamate is released to the synaptic cleft and binds to AMPA and NMDA receptors in postsynaptic neurons. Cell-adhesion proteins NLGN3 and neurexin assist in synaptic formation. Cancer cells can establish functional synapses with neurons by expressing glutamatergic receptors and possibly cell-adhesion proteins to uptake glutamate and induce depolarisation

In neurons, *de novo* formation of functional synapses between neurons and glioma cells has been shown to promote cell growth dramatically [49, 63, 64]. Mechanistically, this is mediated through the secretion of neuroligin-3 (NLGN3) from active neurons—a synaptic protein that sequentially activates the oncogenic PI3K-mTOR signalling pathway and upregulates the proto-oncogene FOS expression, leading to tumour cell proliferation [49, 65]. Moreover, tumour connecting neurons also secrete various neurotransmitters such as neuropeptides, glutamate and serotonin that can further facilitate tumour growth (Fig. 1) [63, 64, 66]. For instance, glioma cells uptake glutamate released by juxtaposed neurons, which acts on glutamatergic receptors located on the tumour cell surface. Several glutamatergic receptors, including α-amino-3-hydroxy-5-methyl-4-isoxazolepropionic acid receptors (AMPARs) and *N*-methyl-d-aspartate receptors (NMDARs), have been found to be expressed on tumour cells (Fig. 1) [66, 67]. By exploiting neuronal signalling, glioma cells trigger an influx of cytoplasmic calcium ions, which propagate through gap junctions, promoting tumour migration and invasion. This is supported by tracking changes of calcium levels and cellular movements in neuron-tumour co-cultures, which show that a higher frequency of calcium signals correlates with greater distances travelled and faster invasion speed of tumour cells [63, 64]. Moreover, Taylor et al. [68] demonstrated that the synapse remodelling factor BDNF promotes malignant synaptic plasticity via its receptor TrkB by facilitating AMPAR trafficking to the glioma cell membrane. In line with this finding, inhibiting TrkB signalling substantially reduces tumour progression and improves survival in DIPG xenograft models. Other predominant neurotransmitters, such as somatostatin, dopamine, and GABA(A), also contribute to gliomagenesis as potent regulators of cancer progression and metastasis [69, 70]. However, the mechanisms by which these factors influence tumour growth, and the tumour developmental stages at which they become critical, remain poorly understood [69, 70]. While researchers have extensively studied how blood vessels, immune cells, and neurons contribute to pHGGs progression, NSCs remain a critical yet underexplored component.

### Functional properties of wild-type neural stem cells

Neurogenesis (formation of neurons) is a tightly regulated process involving proliferation, migration, and differentiation of NSCs into neural progenitor cells (NPCs), followed by terminal differentiation into mature neural cells. This process is critical for establishing cellular architecture and functional neural circuits. While the majority of neurogenesis occurs during embryonic and early postnatal development, NSCs and NPCs persist in the adult brain, retaining the capacity for neurogenesis and gliomagenesis throughout life [71]. Postnatally, NSCs reside within specialised neurogenic niches that provide the necessary microenvironment for their regulation, maintenance, and directed migration to sites of terminal differentiation [72].

**Fig. 2:**
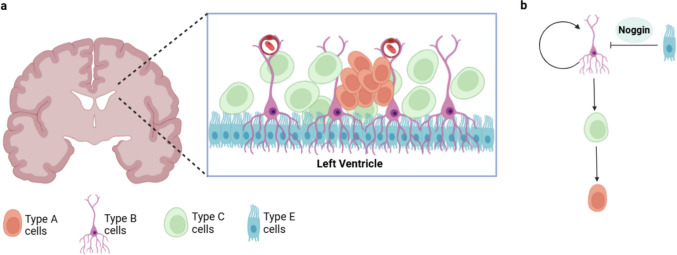
**a** Cellular organisation of the adult mammalian subventricular zone (SVZ). The SVZ, located adjacent to the lateral ventricle (LV), is composed of a layer of ependymal cells (Type E) that line the ventricular surface. Beneath this layer lies a dense population of NSC (Type B), followed by transient amplifying progenitor cells (Type C) and migrating neuroblasts (Type A). **b** Lineage relationships within adult mammalian SVZ. Type B cells divide asymmetrically to either self-renew or give rise to Type C progenitor cells. Type C cells undergo further divisions and differentiate into Type A neuroblasts. Type E cells can secrete noggin to modulate niche signalling by inhibiting excessive neurogenesis and supporting controlled differentiation to Type C and Type A lineages

The largest neurogenic niche in the postnatal brain is the subventricular zone (SVZ) located along the lateral ventricle walls and comprises four primary cell types [73]. Ependymal (Type E) cells form a ciliated lining that facilitates cerebrospinal fluid movement and regulates communication between CSF and interstitial fluid [74]. Behind the layer of Type E cells is a dynamic niche of neural stem cells (Type B), transient amplifying progenitors (Type C), and neuroblasts (Type A) cells that sustain the stem cell population and produce migratory neuroblasts that travel to specific regions of the brain [72] (Fig. 2a) [72]. In particular, Type B cells possess astroglial characteristics and self-renewal capacity, generating highly proliferative intermediate progenitor Type C cells that give rise to young neurons (Type A) through multiple divisions (Fig. 2b) [75, 76]. Type A cells migrate in chains through the rostral migratory stream to the olfactory bulb, where they differentiate into mature interneurons [72, 75, 77]. However, self-renewal of Type B cells can be inhibited by ependymal Noggin secreted from Type E cells to regulate neurogenesis and promote controlled lineage differentiation [75].

Besides the SVZ, wild-type NSCs can also be found throughout adulthood within the hippocampus. Indirect clinical observations suggest that NSCs may contribute to glioblastoma growth and recurrence. Tumours located closer to the SVZ are strongly correlated with higher recurrence rates and poorer patient survival [78]. However, the mechanisms by which NSCs drive glioma progression remain unclear, and whether similar associations exist in pHGGs has not been thoroughly investigated, partly due to limited sample sizes.

In addition to driving tumour growth, NSCs have also been proposed as the cell of origin for pHGGs. Neurogenesis and controlled neuroblast migration are crucial for normal brain development and injury-induced repair responses. Emerging evidence indicates that introducing tumourigenic gene mutations such as p53, PTEN, and EGFR mutations in NSCs can transform gliomagenesis in distal brain regions [79]. Notably, Haag et al. [80] demonstrated that CRISPR/Cas9-mediated introduction of H3K27M mutation, combined with TP53 loss in NSCs, led to oncogenic transformation into tumour cells that recapitulate DIPG. In contrast, deletion of tumour suppressor genes in differentiated neural cells failed to induce malignancy, supporting NPCs may be one of the main cells of origin for gliomas [81]. However, given the complexity of tumourigenic mechanisms and limited study for paediatric gliomas, it remains unclear whether NSCs serve as the cell of origin across all molecular subtypes.

### Glioma stem cells and cellular plasticity in pHGGs

Recent single-cell RNA sequencing studies have revealed molecularly diverse cellular states within pHGGs that resemble genetic signatures of different normal brain cells, including oligodendrocyte (OC-like), astrocyte (AC-like), mesenchymal (MES-like), and oligodendrocyte progenitor (OPC-like) states [18, 82, 83]. This architectural complexity exhibits both inter-tumour heterogeneity (between patients) and intra-tumour heterogeneity (within individual tumours), with diverse cell types forming intricate networks while maintaining unique molecular characteristics that contribute to therapy resistance [84].

Of particular interest is the highly malignant subpopulation of tumour cells that behave like stem cells with extensive self-renewal capacity and differentiation potential, referred to as glioma stem cells (GSCs). GSCs have been proposed as both oncogenic drivers and maintainers of gliomas, playing critical roles in tumour initiation, propagation, and ongoing proliferation [85]. Molecular profiling reveals that neural stem cell markers including SOX2, NESTIN, and CD15 are highly expressed across different pHGG subtypes, with particularly pronounced stem-like signatures in H3-mutant tumours [86]. GSC maintenance involves overexpression of markers including CD109 and hypoxia-induced factors, with HIF-2α maintaining cellular pluripotency within the hypoxic tumour mass [87, 88]. GSCs exhibit remarkable capacity to migrate to distant brain regions through remodelling of the microenvironment via secretion of pro-angiogenic factors such as VEGF, contributing to tumour invasion and recurrence [89, 90].

Critically, emerging evidence demonstrates that these cellular states are not static but exhibit remarkable plasticity, with cells capable of transitioning between differentiation states in response to intrinsic and extrinsic cues. Single-cell analyses reveal that pHGG cells across molecular subtypes can transition between OPC-like, AC-like, and mesenchymal-like states, with these transitions governed by both cell-intrinsic transcriptional programmes—including master regulators such as OLIG2, SOX2, and STAT3—and extrinsic signals from the tumour microenvironment [91–93]. In H3K27M-altered diffuse midline gliomas, dynamic transitions between less-differentiated OPC-like states and more differentiated AC-like states have been extensively characterised, with OPC-like cells demonstrating greater stem-cell properties, enhanced glycolytic metabolism, and increased therapeutic resistance [45, 46, 91]. These state transitions are strongly influenced by hypoxia, metabolic cues, and microenvironmental interactions [46, 91]. H3-wildtype pHGGs exhibit particularly pronounced cellular plasticity with greater transcriptional heterogeneity and more frequent state transitions, potentially reflecting their diverse oncogenic drivers [91, 93]. Plasticity in H3G34-mutant diffuse hemispheric gliomas remains less well-characterised, representing an important area for future investigation.

Neuron-glioma interactions represent a particularly important driver of cellular plasticity across pHGG subtypes. Neuronal activity promotes glioma growth through both synaptic and paracrine mechanisms, with activity-dependent secretion of neuroligin-3 (NLGN3) by glioma cells facilitating formation of glutamatergic synapses with neurons [64, 94]. Synaptic currents drive calcium influx that promotes tumour cell proliferation and shifts cells towards more proliferative, stem-like states [64, 94]. Additionally, neuronal activity stimulates paracrine secretion of growth factors including BDNF, which activates NTRK2 receptors on GSCs, establishing a BDNF–NTRK2 paracrine loop that reinforces proliferative and invasive cellular phenotypes [68, 90]. This neuron-driven plasticity creates a dynamic feedback loop where tumour cells co-opt normal neurodevelopmental programmes to maintain stem-like, therapy-resistant states.

Immune cell interactions similarly drive cellular state transitions in pHGGs. Tumour-associated macrophages and microglia constitute a substantial proportion of the pHGG microenvironment and secrete cytokines including IL-6, IL-10, and TGF-β that influence tumour cell phenotype [95, 96]. These immune-derived signals can induce transitions towards mesenchymal-like states characterised by enhanced invasion, immunosuppression, and therapy resistance. The dynamic interplay between pro-inflammatory and immunosuppressive signals creates a constantly shifting landscape of cellular states within the tumour mass.

Understanding subtype-specific plasticity mechanisms is critical for developing targeted therapeutic strategies, as different molecular contexts may require distinct approaches to disrupt state transitions and overcome therapy resistance. For instance, therapeutic strategies targeting neuron–glioma synapses or metabolic vulnerabilities may be particularly relevant for H3K27M-altered tumours where these mechanisms are well-characterised, while approaches modulating immune cell phenotypes may be broadly applicable across subtypes. Static treatment approaches that target bulk tumour characteristics may inadvertently select for plastic, therapy-resistant cell states, emphasising the need for therapies that account for the dynamic nature of pHGG cellular identity.

### Molecular and functional divergence: distinguishing GSCs from wild-type NSCs

**Fig. 3:**
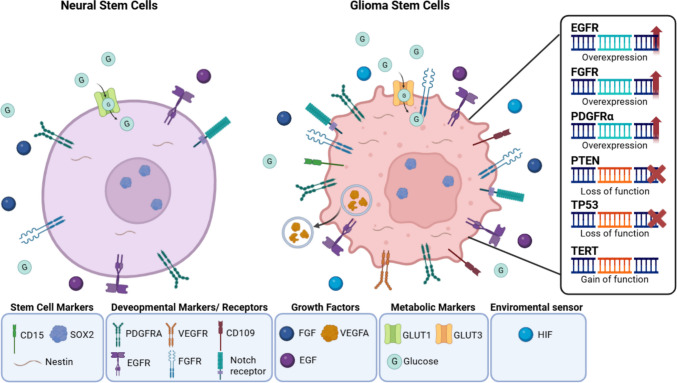
Schematic representation of key genetic and signalling alterations in glioma stem cells compared to neural stem cells in the subventricular zone. Figure was created and adapted from Bakhsinya et al. (2021), with modifications

GSCs and wild-type NSCs share some fundamental capabilities yet differ dramatically in their molecular profiles. While NSCs maintain tightly regulated growth and differentiation, GSCs exhibit hallmarks of cancer traits including self-sustaining growth signals, inhibitory resistance, apoptotic evasive markers, unlimited proliferation, angiogenesis promotion, and invasive behaviour (Fig. 3). At the receptor level, GSCs overexpress critical mitogenic receptors—epidermal growth factor receptor (EGFR) and fibroblast growth factor receptor (FGFR)—that enhance stemness maintenance and self-renewal beyond normal constraints [91, 97, 98]. Similarly, PDGFRA amplification in GSCs drives both hyperplastic growth and radiotherapy resistance, creating treatment challenges in brain cancer [97, 99].

Mutations in tumour suppressor genes fundamentally alter GSC behaviour. TP53 and PTEN mutations disrupt cell cycle regulation and differentiation pathways, with PTEN deficiency profoundly reprogramming NSCs toward GSC-like phenotypes and amplifying oncogenic signalling cascades [100, 101]. Through its regulation of the PI3K/AKT/mTOR pathway, PTEN loss compromises differentiation control and enables apoptotic suppression in GSCs [102]. GSCs also acquire immortality through gain-of-function mutations in the TERT promoter, reactivating telomerase and escaping normal replicative senescence [103, 104]. Their angiogenic capacity exceeds that of NSCs, with receptor tyrosine kinases (EGFR, PDGFR, FGFR) and VEGFA secretion via extracellular vesicles actively promoting vascular development [105–107]. Metabolically, GSCs demonstrate a pronounced Warburg effect, preferentially utilising anaerobic glycolysis and showing greater glucose dependence compared to the more diverse metabolic profile of NSCs [108]. Importantly, these characterisations derive primarily from studies on adult high-grade gliomas. Therefore, caution is warranted when applying these findings to pHGGs, as the molecular and functional landscape of GSCs may differ significantly in paediatric contexts.

### Importance of WT-NPCs and GSCs in glioma development

While GSCs have been routinely demonstrated to promote tumour progression, WT-NPCs may also contribute to glioma growth indirectly. Clinically, gliomas’ proximity to the SVZ is directly correlated with progression, recurrence and lower overall survival in adult glioblastoma patients [109]. Patients with tumours arising closer to the SVZ suffer from significantly higher recurrence rates. The SVZ secretes soluble factors, nutrients, and oxygen essential for NSC maintenance, which may also fuel GSCs in a similar manner to drive glioma growth [110]. However, how exactly do NSCs regulate tumour progression remains underexplored.

Despite the existing NSC and GSC focussed studies in adult gliomas, there is limited data on the involvement of SVZ in pHGGs, largely due to sample size limitations. In light of some clinical data, SVZ invasion was radiologically identified in 53% and 40% of children and adolescents with supratentorial pHGGs in two separate studies, respectively [111, 112]. Gliomas contacting the SVZ exhibit a distinct proteomic profile compared to tumours contacting other brain regions, possibly due to excessive growth factor expression, accelerating glioma progression [113]. These alterations may stem from SVZ NSCs’ secretion of chemoattractant complex, such as pleiotrophin and its binding partners, that promotes tumour invasion towards the lateral ventricle by activating the Rho/Rho-associated protein kinase signalling pathway in DIPG cells [114]. Rho GTPases are involved in all stages of cancer progression, function as key regulators of the cytoskeleton to facilitate tumour cell migration and modulate gene transcription to support tumour cell survival and interaction with the surrounding stromal cells [115]. Furthermore, glioblastoma-derived extracellular vesicles can transform NSCs into a highly proliferative and migratory GSCs-like phenotype. This transformation is mediated through the dysregulation of key gene signalling such as CD44, SCSL14 and HMGA1, as well as activation of the PI3K-mTOR pathway in the recipient NSCs, thereby contributing to tumour progression and recurrence [116, 117]. These findings support the ability of glioma cells to invade the SVZ and suggest phenotypic and epigenetic modulation by the SVZ microenvironment.

Taken together, both WT-NPCs and GSCs can indirectly influence each other, contributing to glioma initiation, progression and migration. Thus, regulating their signalling pathways is crucial for stem cell survival and tumour development. While key pathways such as TP53, MET kinases and AKT have been widely studied, metabolic signalling pathways in gliomas remain underexplored. Here, we will discuss a few emerging metabolic pathways hijacked by gliomas to promote metabolism adaptation.

## Preclinical models for pHGG research

The development of effective therapies for pHGGs has been significantly hindered by the historical lack of adequate preclinical models. Early therapeutic strategies were largely extrapolated from adult high-grade glioma research, which proved ineffective given the fundamental molecular and developmental differences between paediatric and adult tumours [47]. The reintroduction of safer biopsy techniques and expanded autopsy programmes has provided unprecedented access to pHGG tissue, catalysing the development of more sophisticated and clinically relevant experimental models [118, 119]. Understanding these models is essential for interpreting preclinical findings and translating discoveries into clinical applications.

### Patient-derived xenograft models

Patient-derived xenograft (PDX) models have emerged as the most critical tools for studying pHGG biology and evaluating novel therapeutics. Unlike traditional cell line-based approaches, PDX models preserve the molecular heterogeneity and histopathological features of original patient tumours. Initial challenges in establishing DIPG cell lines were overcome through neurosphere culture systems using neural stem cell conditions from autopsy specimens. These neurospheres express varying levels of stem cell markers including Nestin, GFAP, Sox2, Olig2, and CD133, reflecting their primitive neural progenitor phenotype. Stereotactic transplantation into the fourth ventricle or directly into the pons of immunodeficient mice generates tumours that diffusely infiltrate the brainstem, cerebellum, and cerebrum with histopathology reminiscent of human DIPG [118, 119].

Recent comprehensive efforts have substantially expanded the available pHGG PDX models. Phoenix et al. [120] established 21 patient-derived orthotopic xenograft models representing diverse molecular subgroups of pHGG, including H3K27M-mutant DIPG, H3G34R/V-mutant diffuse hemispheric gliomas (DHG), and H3-wildtype variants. Detailed molecular characterisation demonstrated that these models faithfully recapitulate the histopathology, DNA methylation signatures, mutational profiles, and gene expression patterns of their corresponding patient tumours. These models have proven valuable for high-throughput drug screening and *in vivo* validation of therapeutic efficacy, with *in vitro* screening results predicting variable *in vivo* responses to PI3K/mTOR and MEK pathway inhibitors [120].

Despite their utility, PDX models exhibit important limitations. The use of immunodeficient host mice precludes investigation of tumour-immune interactions, a critical consideration given the growing interest in immunotherapeutic approaches. Additionally, establishment rates vary by tumour grade and molecular subtype, with particularly low success rates for IDH-mutant gliomas [121]. Methodological variability across laboratories—including differences in host mouse strain, injection location, cell number, and suspension matrices—can significantly impact tumour growth kinetics and invasiveness, underscoring the need for standardised protocols [120].

### Genetically engineered mouse models

Genetically engineered mouse models (GEMMs) offer complementary advantages by enabling the study of tumourigenesis in its native developmental and anatomical context with an intact immune system. GEMMs are particularly valuable for investigating the cell of origin for pHGGs, the sufficiency of specific genetic alterations to drive tumour formation, and the sequential roles of oncogenes and tumour suppressors in gliomagenesis [122].

Multiple sophisticated approaches have been developed to generate pHGG GEMMs. The RCAS (replication-competent avian sarcoma-leukosis virus)/TVA system utilises retroviral gene delivery to cells expressing the avian TVA receptor under cell-type specific promoters. Early RCAS-based brainstem glioma models demonstrated that targeting neural progenitor cells in the developing pons with PDGF-B overexpression combined with p53 loss could generate brainstem gliomas. Importantly, these models provided the first demonstration that the CDK4/6 inhibitor PD-0332991 could prolong survival beyond radiation therapy alone, highlighting the translational potential of GEMMs [123].

A critical finding from H3K27M-incorporating models was that this mutation alone is insufficient to drive tumour formation. Multiple groups demonstrated that transformation of neural progenitor cells requires H3K27M in combination with additional genetic alterations, most commonly TP53 loss and activation of PDGF receptor signalling [80, 124, 125].These findings were achieved using advanced genetic engineering techniques that allow researchers to introduce specific mutations into developing brain cells at precise timepoints. For instance, some models use DNA delivery systems that insert the H3K27M mutation directly into neural progenitor cells as they develop in the brainstem. Others use genetic switches that can be turned on or off to activate mutations in specific cell types at chosen developmental stages [124, 125].

A major limitation of traditional GEMMs has been the absence of a functional immune system, preventing researchers from testing immunotherapies. Recent innovations have overcome this challenge by creating models that develop tumours in mice with intact immune systems while simultaneously expressing human tumour proteins that can be targeted by immune-based therapies [126]. These immunocompetent models now enable preclinical testing of CAR T-cell therapies—a promising immunotherapy approach where a patient’s immune cells are engineered to recognise and attack tumour cells—in a setting that more closely mimics the human disease environment [126].

### Emerging immunotherapeutic approaches in pHGGs

Despite decades of effort, conventional therapies for pHGGs have yielded minimal survival improvements, necessitating novel therapeutic strategies. Immunotherapy represents a promising frontier, with CAR T-cell therapies and oncolytic viruses emerging as particularly encouraging approaches in early-phase clinical trials.

CAR T-cell therapies involve genetically engineering patients’ T lymphocytes to express chimeric antigen receptors targeting tumour-associated antigens. Three targets have demonstrated particular promise in pHGGs: disialoganglioside GD2, B7-H3 (CD276), and human epidermal growth factor receptor 2 (HER2), all of which are highly expressed in paediatric diffuse midline gliomas and other pHGG subtypes [127–130]. GD2-targeting CAR T-cells delivered intravenously or intracerebroventricularly have shown remarkable activity in H3K27M-mutant diffuse midline gliomas, with 9 of 11 patients experiencing neurological improvement and 7 demonstrating tumour shrinkage in a recent phase I trial [129, 130]. Notably, some patients transitioned from wheelchair-bound to ambulatory within weeks of treatment [131]. B7-H3-targeted CAR T-cells administered intracerebroventricularly in children with DIPG achieved a median overall survival of 19.8 months, with three patients surviving over 40 months from diagnosis—substantially exceeding historical outcomes [128]. HER2-directed CAR T-cells have similarly demonstrated feasibility and preliminary efficacy in early trials [127]. However, significant challenges remain, including blood–brain barrier penetration, immunosuppressive tumour microenvironment, antigen heterogeneity, and cytokine release syndrome [127, 128, 130].

Oncolytic viruses selectively replicate in and destroy tumour cells while stimulating antitumour immune responses. The oncolytic adenovirus DNX-2401 (tasadenoturev, Delta-24-RGD) administered intratumorally followed by radiotherapy in newly diagnosed DIPG patients achieved a median overall survival of 17.8 months, with tumour size reduction observed in 9 of 12 patients and conversion of immunologically “cold” tumours to “hot” through enhanced T-cell infiltration [131, 132]. Similarly, genetically engineered herpes simplex virus-1 G207 demonstrated acceptable safety and evidence of clinical responses in recurrent paediatric high-grade gliomas, with a median overall survival of 12.2 months and transformation of immunologically silent tumours into inflamed, lymphocyte-rich environments [133, 134]. These virotherapies appear particularly well-suited to paediatric brain tumours, which exhibit enhanced sensitivity to viral oncolysis compared to adult glioblastomas [134].

While both CAR T-cell therapies and oncolytic viruses remain investigational, early clinical data suggest meaningful therapeutic potential. Future strategies will likely involve combination approaches—integrating these immunotherapies with conventional treatments or combining CAR T cells with oncolytic viruses—to overcome current limitations and achieve durable responses in this devastating disease.

## Metabolic reprogramming in glioma cells

### Glucose metabolism in glioma cells

Glucose metabolism is vital in glioma development, driven by metabolic reprogramming and intricate molecular mechanisms. Understanding these processes is essential for developing novel therapies targeting glucose metabolism.

The “Warburg effect” is a hallmark defined as a metabolic shift in gliomas, where affected cells exhibit an increase in glucose uptake for glycolysis. Aerobic glycolysis, the oxygen-dependent conversion of glucose to lactate, is the core metabolic pathway that provides the energy and essential macromolecules, such as lipids, nucleic acids, and proteins, necessary for glioma proliferation and progression [135, 136]. A recent plasma metabolomic analysis of 60 glioma patient samples revealed elevated glycolytic metabolites—glucose, lactate, and pyruvate—and reduced glutamate and TCA cycle intermediates, including citrate and succinate [137]. Particularly, under oxygen-rich conditions, glioma cells favour glycolysis to convert glucose to lactate, a process that yields less energy but supports the rapid proliferation of tumour cells. Moreover, glioma cells exhibited increased levels of lactate dehydrogenase (LDH), which catalyses the conversion of pyruvate to lactate [138]. The accumulation of lactate fosters an acidic microenvironment that enhances tumour invasion, promotes neovascularisation, and leads to immunosuppression [138]. Additionally, glioma-derived lactate is taken up by surrounding cells and converted into pyruvate, then reintroduced into the energy cycle, further driving tumour growth and metastasis [108]. This metabolic feature is further supported by Autry et al. [139] using carbon-13 metabolic imaging to demonstrate elevated LDH levels and lactate in lesions in DIPG xenografts.

Tumour microenvironmental conditions play a key role in modulating tumour glucose metabolism. For instance, physiological stressors including hypoxia and nutrient deprivation drive GLUT upregulation to sustain the energy demands of proliferating tumour cells. The alpha subunit of Hypoxia-inducible factor 1 (HIF-1 $\alpha$) is overexpressed in several human cancers, including gliomas, where it enhances GLUT expression during hypoxia to increase glucose uptake and adapt to oxygen availability [140]. Moreover, an increasing number of studies report that glucose transporter proteins GLUT1 and GLUT3 are overexpressed in gliomas. Specifically, GSCs exhibit high levels of GLUT3 expression, which strongly correlates with poor patient survival. Both *in vitro* and *in vivo* studies have shown that inhibiting GLUT3 expression significantly impairs tumour growth by limiting glucose uptake and reducing glycolytic activity. The neuronal glucose transporter GLUT3 has a five-fold higher affinity for glucose than GLUT1 and other isoforms and is largely restricted to cells with high glucose demand in a glucose-poor microenvironment [48, 141]. The upregulation of GLUT3 represents a key oncogenic adaptation, enabling GSCs to outcompete surrounding healthy cells for glucose, thereby preserving their function and survival [141]. This enhanced glucose acquisition mechanism provides GSCs a critical metabolic advantage within the nutrient-deprived microenvironment.

Furthermore, glioma cells upregulate multiple other glucose metabolism-related proteins, including phosphofructokinase (PFK1), pyruvate dehydrogenase lipoamide kinase isozyme 1 (PDK1), hexokinase II (HK2), alpha-enolase (ENO1) and gamma-enolase (ENO2) to reinforce their glucose metabolism reprogramming. Under hypoxic conditions, glioma cells upregulate PDK1, which inhibits pyruvate dehydrogenase and restricts pyruvate entry into the TCA cycle, shifting energy production from oxidative phosphorylation to glycolysis [142]. Notably, multiple animal studies have demonstrated that suppressing PDK1 and other key proteins such as HK2 and PFK1 significantly inhibits tumour growth [143, 144]. Collectively, these findings underscore the central role of glucose metabolism reprogramming in glioma progression, particularly in adapting to the harsh tumour microenvironment.

### Lipid metabolism in glioma

Lipid metabolism alterations observed in glioma cells are increasingly recognised as critical drivers of tumour initiation and progression regulated by oncogenic signalling pathways [145]. The dysregulated lipid metabolism in gliomas primarily impacts free fatty acids and cholesterol, which are crucial for tumour growth and contribute to therapy resistance. While studies on lipid metabolism alteration in adult glioma have been extensively documented, specific investigations into pHGGs are limited.

High-grade glioma cells significantly increase fatty acid (FA) uptake to sustain membrane biosynthesis during rapid growth and proliferation [8]. The activation of metabolic pathways such as PI3K/Akt/mTOR and AMPK pathway indirectly enhances lipogenesis and FA utilisation by regulating FA transport proteins and enzymes involved in FA oxidation [146]. For instance, the PI3K/Akt/mTOR pathway promotes lipogenesis through the mTORC1 and mTORC2 complexes. The mTORC1 enhances the activity of the lipogenic transcription factor SREBP-1, driving the expression of key lipogenic enzymes including ATP citrate lyase (ACLY) and fatty acid synthase (FASN). Meanwhile, mTORC2 supports lipogenesis through both AKT-dependent and independent pathways that further modulate SREBP-1 activity [146]. Moreover, fatty acid synthesis can also influence GSC metabolism. Hale et al. [147] showed that GSCs have increased levels of the FA scavenger receptor CD36 to facilitate exogenous uptake of FA which promotes enhanced self-renewal capacity and enhanced tumour growth *in vivo*.

Another metabolic alternation in DIPG is cholesterol biosynthesis. Cholesterol is essential for various physiological processes, serving as a key component in lipoprotein formation, a precursor for steroid hormones and bile acids, and a major component of synaptic vesicles in the CNS [148, 149]. RNA sequencing data showed that genes such as HMG-CoA reductase and Farnesyl-Diphosphate Farnesyltransferase 1 (FDFT1), which encode cholesterol biosynthetic enzymes, are upregulated in DIPG cells [46, 150]. SREBP2, a critical membrane-bound transcription factor, governs cholesterol synthesis by enhancing expression of HMG-CoA reductase and LDL receptor genes [151]. In adult HGGs, SREBP2 exhibits significant upregulation, particularly in tumours harbouring EGFR mutations and PI3K pathway disruptions [152]. For instance, Gu et al. [153] reported that SREBP2 expression is elevated in the glioblastoma core compared to the invasive margins. They also found that SREBP2 supports cholesterol biosynthesis in GSCs and drives cholesterol-dependent oncogenic mechanisms, promoting tumour growth and proliferation under starvation. Hence, SREBP2 may play a similar role in pHGGs, providing insight into tumour biology and representing a potential avenue for future research. Most significantly, clinical data suggest that SREBP2 upregulation can serve as a potential prognostic marker for diffuse gliomas. Whether it can be leveraged as a therapeutic target in future treatment strategies remains to be explored [154].

Cholesterol homeostasis in cells is maintained through a tightly regulated balance of *de novo* synthesis, uptake from circulation, storage as esters, and efflux via transporters. In glioma cells, cholesterol efflux is mediated by Liver X Receptors (LXRs), which upregulate ATP-binding cassette transporters’ activity and simultaneously downregulate LDL receptor expression [155]. This coordinated regulatory mechanism maintains optimal cholesterol levels essential for tumour cell function. In addition, cholesterol metabolism products, such as oxysterols, are elevated in gliomas and influence immunity, inflammation, and tumour development [156]. Dysregulated cholesterol metabolism activates inflammatory mediators like IL-1β, TNF-α, and IL-6, altering immune responses in gliomas. These inflammatory factors can, in turn, modulate cholesterol metabolism by affecting cholesterol synthases and transport proteins, impacting tumour progression [157].

Overall, lipid metabolism in gliomas encompasses intricate interactions between the synthesis, regulation, and utilisation of fatty acids and cholesterol, with their metabolic byproducts further shaping the distinct tumour biology.

### Metabolic plasticity of GSCs

**Fig. 4:**
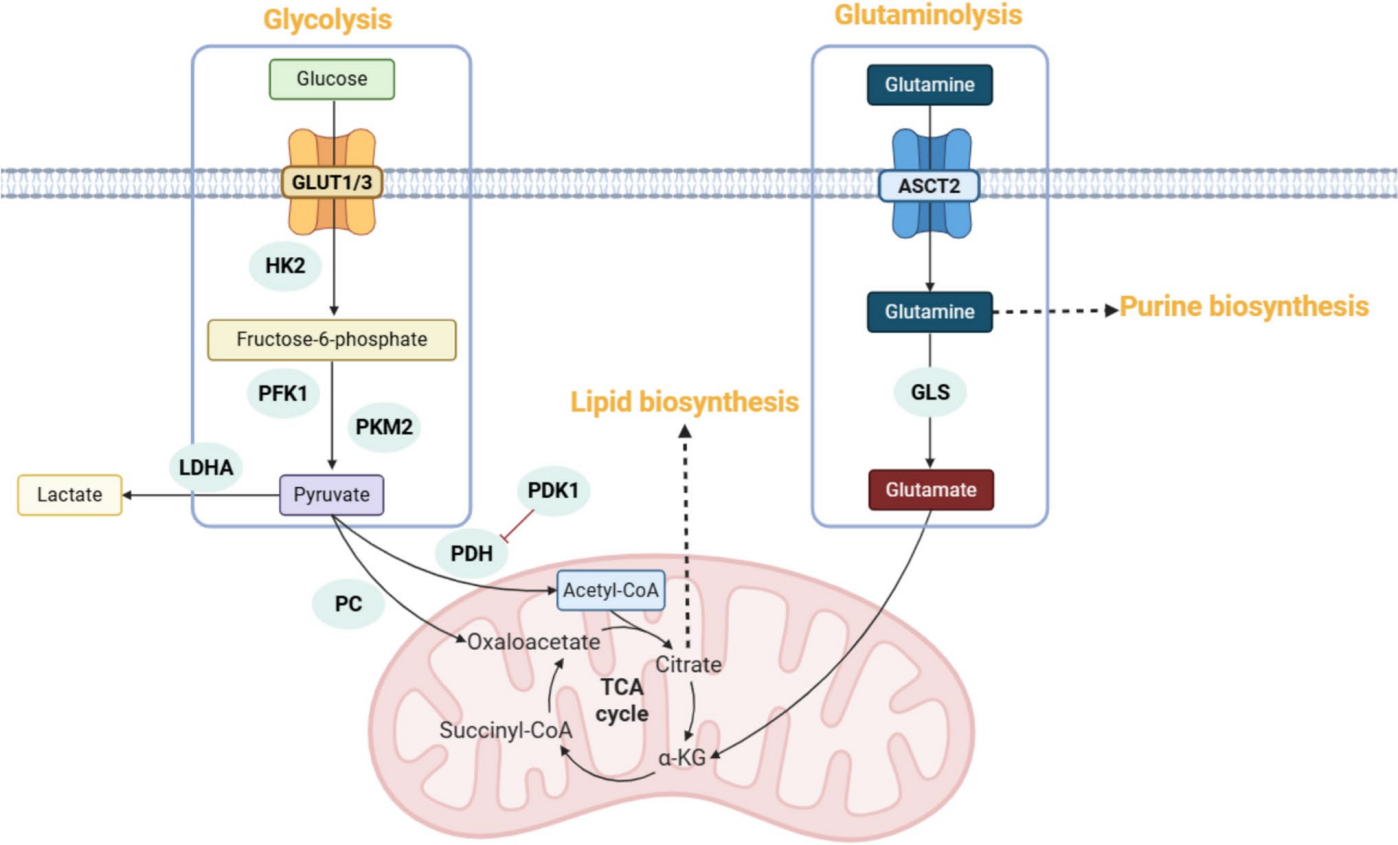
Metabolic pathway adaptations in glioma stem cells (GSCs). Glucose is imported into the cytoplasm via GLUT1 and/or GLUT3 transporters and undergoes glycolysis to produce pyruvate. This process is regulated by key glycolytic enzymes, including HK2, PFK1, PKM2, LDHA, and PDK1. Pyruvate enters the mitochondria and contributes to the tricarboxylic acid (TCA) cycle by being converted to oxaloacetate via pyruvate carboxylase (PC) or to acetyl-CoA via pyruvate dehydrogenase (PDH). The TCA cycle intermediate citrate can exit the mitochondria to support lipid biosynthesis. Glutamine is imported via the ASCT2 transporter and either contributes to purine biosynthesis or is converted to glutamate by glutaminase (GLS) to fuel the TCA cycle

Emerging research has revealed profound metabolic reprogramming in glioma stem cells (GSCs), demonstrating the ability of GSCs to hijack and rewire fundamental metabolic pathways to sustain their aggressive growth (Fig. 4). GSCs exhibit distinct metabolic profiles characterised by exceptional plasticity—a critical adaptation that supports their survival under challenging conditions and promotes the induction of stem-like phenotypes in surrounding tumour bulk cells, thereby driving disease progression and therapeutic resistance [158]. The metabolic process in GSCs is highly complex and heterogeneous. Notably, a study on oxygen consumption rate and external acidification rate of GSCs found that GSCs neurospheres exhibited lower glucose uptake, reduced lactate production and higher ATP levels, suggesting a preference for oxidative phosphorylation to maximise energy output [159]. Metabolic profiling reveals that pyruvate carboxylation is a major metabolic pathway in GSCs [160]. Within the mitochondria, glycolytic pyruvate is converted to either acetyl-CoA by pyruvate dehydrogenase or oxaloacetate by pyruvate carboxylase for glucose metabolism. Intriguingly, pyruvate carboxylase activity is elevated in GSCs, and its inhibition can dampen the self-renewal ability and increase the differentiation marker expressions [160]. This highlights pyruvate carboxylase as a crucial regulator of GSCs’ survival and stemness maintenance. Interestingly, some studies observed no increase in ATP levels but enhanced lactate production and glucose uptake in GSCs, indicating glycolytic dominance [161]. This discrepancy may stem from GLUT3 upregulation and voltage-dependent anion channel 2 (VDAC2) downregulation, which promote glycolysis via phosphofructokinase (PFKP) while preserving GSC survival and stem-like features [48, 162].

Metabolic heterogeneity within a single tumour has been well documented, with slow-cycling GSCs predominantly relying on oxidative phosphorylation while fast-cycling GSCs preferentially utilise aerobic glycolysis, conferring chemoresistance and invasiveness [163]. Analysis of variant allele frequencies in paediatric high-grade gliomas (pHGGs) has revealed that slow-cycling GSCs give rise to rapidly proliferating progenitor-like cells and are responsible for tumour recurrence [164]. GSCs exhibit remarkable metabolic plasticity, dynamically regulating glycolytic enzymes such as HK2, PKM2, LDHA, and PDK1 to shift between glycolysis and oxidative phosphorylation in response to environmental changes, ensuring sustained biosynthesis [165]. Taken together, these findings highlight the remarkable metabolic heterogeneity and adaptability of GSCs, which not only sustain their stemness and promote tumour growth but also pose significant challenges to treatment. Beyond these metabolic adaptations, the interplay between metabolism and epigenetic regulation represents an additional layer of complexity that fundamentally shapes pHGG behaviour.

### Metabolic-epigenetic crosstalk in pHGGs

Metabolic reprogramming and epigenetic regulation are intimately interconnected in pHGGs, with metabolites serving as essential cofactors and substrates for chromatin-modifying enzymes that shape the transcriptional landscape and influence tumour behaviour [166–168]. This metabolic-epigenetic crosstalk represents a fundamental mechanism through which cellular metabolism directly regulates gene expression, cellular plasticity, and disease progression across pHGG molecular subtypes.

Central to this crosstalk are key metabolites that function as obligate cofactors for chromatin-modifying enzymes. α-ketoglutarate (α-KG), a TCA cycle intermediate, serves as an essential cofactor for TET DNA demethylases and JmjC domain-containing histone demethylases [29, 169, 170]. As previously discussed, H3K27M-altered diffuse midline gliomas exploit this relationship by enhancing glycolytic and glutaminolytic metabolism to maintain elevated α-KG levels, which sustain the characteristically low H3K27me3 state that defines these tumours [43]. Conversely, IDH-mutant gliomas accumulate the oncometabolite 2-hydroxyglutarate (2-HG), a structural analogue of α-KG that competitively inhibits α-KG-dependent dioxygenases [29]. This inhibition results in widespread DNA and histone hypermethylation—termed the glioma-CpG island methylator phenotype (G-CIMP)—fundamentally altering the epigenetic landscape and blocking cellular differentiation [28, 171, 172].

Additional metabolites critical to this crosstalk include acetyl-CoA, which serves as the substrate for histone acetyltransferases that catalyse histone acetylation associated with transcriptionally active chromatin, and S-adenosylmethionine (SAM), the universal methyl donor for DNA and histone methyltransferases [173–175]. Fluctuations in these metabolites, driven by alterations in glycolysis, fatty acid oxidation, and one-carbon metabolism, directly influence chromatin modifications and gene expression programmes.

This metabolic-epigenetic crosstalk has profound implications for cellular plasticity and therapeutic resistance in pHGGs. Metabolically driven epigenetic changes can facilitate transitions between cellular states, promoting tumour adaptation to therapeutic pressures. Moreover, the dependence of epigenetic enzymes on metabolic cofactors creates therapeutic vulnerabilities—for instance, depleting α-KG pools in H3K27M tumours through metabolic inhibition may indirectly modulate the epigenetic landscape and restore differentiation programmes [43]. Understanding this bidirectional relationship between metabolism and epigenetics is essential for developing integrated therapeutic strategies in pHGGs.

## Conclusion

Glioma stem cells are the key drivers of paediatric high-grade gliomas, fuelling tumour growth, spread, and treatment resistance through their remarkable ability to adapt and survive. These cancer stem cells exhibit exceptional metabolic flexibility, switching between different fuel sources—glucose, fats, and amino acids—to maintain themselves under changing conditions within the tumour environment. Importantly, the metabolic changes in these tumours are closely linked to their epigenetic abnormalities: metabolites produced from altered metabolism directly influence how genes are turned on or off. For example, in H3K27M-altered gliomas, elevated α-ketoglutarate levels help maintain the characteristic low H3K27me3 state that defines these tumours, while in IDH-mutant gliomas, accumulation of 2-hydroxyglutarate causes widespread changes in DNA methylation. This intimate connection between metabolism and gene regulation creates potential therapeutic vulnerabilities—blocking key metabolic pathways can alter the epigenetic landscape and disrupt glioma stem cell maintenance. However, the inherent adaptability of these stem cells allows them to quickly adjust their metabolism and shift between different cellular states to evade therapy. Effective treatment will require combination approaches that simultaneously target multiple metabolic pathways, disrupt epigenetic regulation, and eliminate the stem cell population. Moving forward, developing improved models that better recapitulate paediatric tumour biology and understanding how stem cell plasticity enables therapeutic escape will be essential for designing successful treatments that exploit the unique metabolic and epigenetic vulnerabilities of each pHGG subtype.

## Data Availability

No datasets were generated or analysed during the current study.
